# Multi-Objective Optimization of Adhesive Joint Strength and Elastic Modulus of Adhesive Epoxy with Active Learning

**DOI:** 10.3390/ma17122866

**Published:** 2024-06-12

**Authors:** Paripat Kraisornkachit, Masanobu Naito, Chao Kang, Chiaki Sato

**Affiliations:** 1 Data-Driven Polymer Design Group, Research Center for Macromolecules and Biomaterials, National Institute for Materials Science (NIMS), Ibaraki 305-0047, Japan; kraisornkachit.paripat@nims.go.jp; 2 Program in Materials Science and Engineering, Graduate School of Pure and Applied Sciences, University of Tsukuba, Ibaraki 305-8577, Japan; 3 Institute of Innovative Research (IIR), Tokyo Institute of Technology, Kanagawa 226-8503, Japan; kang.c.ab@m.titech.ac.jp (C.K.); csato@pi.titech.ac.jp (C.S.)

**Keywords:** epoxy, adhesive, elastic modulus, machine learning, active learning, experimental testing, multi-objective optimization, sandwich-structured material

## Abstract

Studying multiple properties of a material concurrently is essential for obtaining a comprehensive understanding of its behavior and performance. However, this approach presents certain challenges. For instance, simultaneous examination of various properties often necessitates extensive experimental resources, thereby increasing the overall cost and time required for research. Furthermore, the pursuit of desirable properties for one application may conflict with those needed for another, leading to trade-off scenarios. In this study, we focused on investigating adhesive joint strength and elastic modulus, both crucial properties directly impacting adhesive behavior. To determine elastic modulus, we employed a non-destructive indentation method for converting hardness measurements. Additionally, we introduced a specimen apparatus preparation method to ensure the fabrication of smooth surfaces and homogeneous polymeric specimens, free from voids and bubbles. Our experiments utilized a commercially available bisphenol A-based epoxy resin in combination with a Poly(propylene glycol) curing agent. We generated an initial dataset comprising experimental results from 32 conditions, which served as input for training a machine learning model. Subsequently, we used this model to predict outcomes for a total of 256 conditions. To address the high deviation in prediction results, we implemented active learning approaches, achieving a 50% reduction in deviation while maintaining model accuracy. Through our analysis, we observed a trade-off boundary (Pareto frontier line) between adhesive joint strength and elastic modulus. Leveraging Bayesian optimization, we successfully identified experimental conditions that surpassed this boundary, yielding an adhesive joint strength of 25.2 MPa and an elastic modulus of 182.5 MPa.

## 1. Introduction

In the realm of materials science and engineering, the study of adhesive materials plays a pivotal role in advancing technologies across diverse industries [1], for example, automotive [2], aerospace [3] and construction materials [4]. The adhesive joint strength, representing the force required to break or deform a bonded interface, and the elastic modulus, signifying a material’s resistance to deformation under applied stress, are two fundamental properties that have important influence on the performance and reliability of adhesive systems. The adhesive joint strength serves as a crucial metric in assessing the integrity of bonded structures. A robust and durable bond is often synonymous with high adhesive joint strength, ensuring the stability of assemblies in various environments and under different loading conditions [5]. However, this strength is not isolated from the elastic modulus of the adhesive material. The elastic modulus, or stiffness, defines how the material responds to external forces [6], influencing the distribution of stresses within the adhesive joint. As such, the interplay between adhesive joint strength and elastic modulus becomes a critical factor in determining the overall performance and longevity of bonded structures [7]. Understanding the trade-offs and synergies between adhesive joint strength and elastic modulus is imperative for optimizing material selection based on specific application requirements. The relationship between adhesive joint strength and elastic modulus is often complex. Several studies have reported that high-elastic-modulus materials have a higher adhesive joint strength [8,9]. However, lower-modulus adhesives can provide the ability to absorb external forces, and this ability is an important factor in adhesive materials used in bonding parts that are easily broken or damaged [7]. In architectural applications like structural glazing systems, low elastic adhesives are used to bond glass panels to the structural framework. These low modulus adhesives can handle high stress gradients at glass interfaces [10].

Achieving an optimal balance between these properties is a multifaceted challenge, as conventional optimization approaches often focus on a singular property, potentially neglecting the complex interdependencies that exist. Multi-objective optimization presents a paradigm shift by simultaneously addressing the enhancement of many properties. Recently, high productivity of heat-resistant epoxy matrix systems was successfully achieved using multi-objective optimization along with machine learning. This accomplishment has never been achieved, even though the conventional trial-and-error experiment has been attempted up to three hundred times [11]. Furthermore, because the required properties frequently conflict with one another in nature, multi-objective optimization can be a candidate method from the material design point of view. The maximum molecular weight and the maximum of number average degree of branching of polymers were achieved in the polymerization process with the assistance of a multi-objective optimization approach. This can reduce unnecessary costs and time consumption in the experimental stage [12]. Moreover, the multi-objective optimization approach was applied in optimizing material removal rate and taper during electrochemical discharge machining of the silicon carbide-reinforced epoxy composites, and it was reported that there was a 15% improvement in taper reduction, together with the material removal rate decreasing by 0.91%. In addition, utilizing multi-objective optimization is able to address the trade-off problem [13]. This approach not only extends the edge of discovery but also accelerates the study process for humankind. 

The adhesive joint strength has been measured previously by using single-lap shear joints [14]. On the other hand, indentation hardness measurements were used to evaluate the elastic modulus of the same adhesives, owing to the significant convenience in sample preparation and measurements compared to the widely used tensile test. The relationship between hardness and elastic modulus has been thoroughly studied, and several methods for calculating elastic modulus from hardness measurements have been reported previously [15]. Nevertheless, before measuring the hardness of polymeric specimens, it is important to prepare appropriate samples to ensure accurate and reliable results. The polymeric specimens have to be prepared in standardized shapes and sizes suitable for the specific hardness testing method being used. Additionally, the surface of the specimens must be prepared to ensure flatness and smoothness, as irregularities or rough surfaces can directly affect hardness measurements. Moreover, voids and bubbles lead to inaccurate results; thus, homogeneity of the polymer material is one of the important factors. 

The adhesive joint strength and elastic modulus are key mechanical properties that directly influence the adhesion performance of adhesive materials. However, simultaneously studying multiple properties can be time-consuming and costly. In this work, we propose to investigate the adhesive joint strength and elastic modulus of epoxy adhesives using a multi-objective optimization approach. A metal mold is designed and introduced to prepare the polymeric specimens according to the French standard NFT 76-142 [16] to eliminate porosity. The elastic modulus of the polymeric specimens is calculated according to Shore hardness which is measured by two types of durometers referring to ASTM-D2240 [17]. The experimental conditions are designed and conducted to obtain a small initial dataset of the first 32 conditions. The machine learning model is trained and validated for optimizing accuracy. Then, prediction of the extended 256 conditions is carried out together with the active learning method. Active learning is a strategy that can be employed to improve model accuracy and reduce deviations in predicted results. Active learning is a machine learning approach that involves selecting the most informative data points for labeling or further training, with the goal of enhancing the model performance while minimizing the amount of labeled data needed. After that, the trade-off boundary is obtained after the improved prediction of the 256 conditions. Finally, Bayesian optimization is employed to identify experimental conditions with predicted results that can overcome the trade-off boundary. The results are confirmed by checking the actual experiments. Adhesive epoxies with desired adhesive joint strength and elastic modulus properties can be fabricated with the provided conditions from the proposed multi-objective optimization approaches.

## 2. Materials and Methods

### 2.1. Experiments

#### 2.1.1. Materials

**Figure 1:**
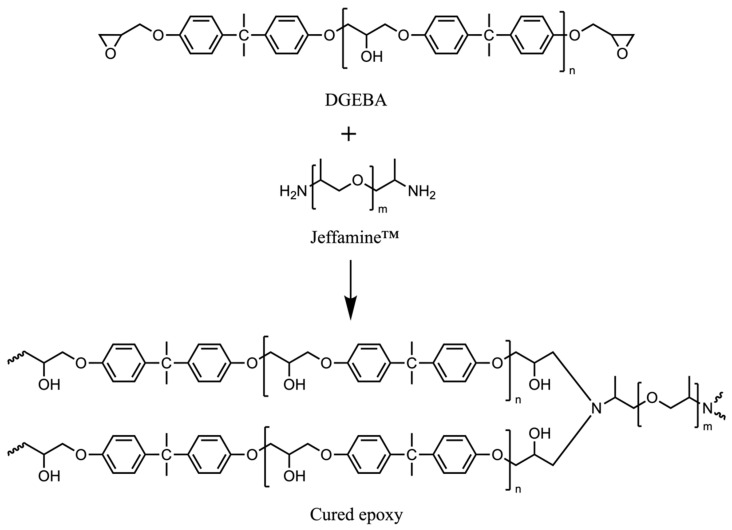
Chemical structures of DGEBA, Jeffamine™ and cured epoxy.

In this study, a commercial epoxy resin, Diglycidyl ether of bisphenol A-based epoxy resin (DGEBA) from Mitsubishi Chemical Corporation, Tokyo, Japan, with 4 different molecular weights, and a commercial diamine curing agent (Jeffamine™), Poly(propylene glycol) bis(2-aminopropyl ether) from Sigma-Aldrich, Tokyo, Japan, with 4 different molecular weights, were used. The molecular weights of DGEBA and Jeffamine™ are *M*_wE_ = {370, 1650, 2900 and 3800} g/mol and *M*_wC_ = {230, 400, 2000 and 4000} g/mol, respectively. The appearance of DGEBA with *M*_wE_ of 230 g/mol is in liquid phase; in contrast, it is in solid phase for *M*_wE_ of 1650, 2900 and 3800 g/mol. Additionally, the appearance of Jeffamine™ with *M*_wC_ of 230 and 400 g/mol is viscous liquid; on the other hand, it is a highly viscous liquid for 2000 and 4000 g/mol. All chemicals were used as received without further purification or pretreatment. Generally, the stoichiometric mixing ratio of epoxy resin is two moles of epoxy resin per one mole of curing agent. The chemical structures and reactions of DGEBA and Jeffamine™ are illustrated in Figure 1.

#### 2.1.2. Preparation of Specimens

A metal mold was designed and fabricated specifically for this study according to the French standard NFT76-142 [16]; the illustration is shown in Figure S1 (Supplementary Materials). In principle, the objective of using this mold is not only to produce cured adhesive specimens with a flat and smooth surface according to the standard but also to remove the voids and bubbles entrapped inside the specimens as much as possible. This metal mold consists of a metal lid and a metal base, and the adhesive specimen is fitted into a 6 mm thick silicon rubber frame (obtained from Axel 4-1371-08) at the center of the mold. This silicon rubber is bordered by a metal box which contains 4 small gaps on the topside of each edge; these gaps allow the adhesive mixture to overflow during high-pressure curing conditions. An overflow of adhesive mixture is potentially able to remove gas bubbles entrapped in the specimens [18]. This mold created a square-shaped specimen with a dimension of 40 × 40 × 6 mm^3^ according to the standard test method for rubber property ASTM-D2240 [17]; the illustration is shown in Figure S1. Teflon tape (obtained from Axel 3-5579-09) was pasted onto the metal lid and metal base on the contact side of the adhesive specimen in order to reduce the difficulty in removing the cured adhesive specimens from the mold.

DGEBA and Jeffamine™ were separately preheated in the oven at 190 °C for 30 min; this process is to ensure that solid epoxy is melted into liquid prior to the mixing step. After that, it was rapidly mixed together in the disposal bottle glass for a couple seconds until the mixture achieved a homogenous phase, and then, the mixture was poured into a metal mold. In total, four specific amine-to-epoxide ratios were investigated in this study: *r* = {0.75, 1.00, 1.25 and 1.50}. For example, *r* = 1 represents the stoichiometric mixing ratio of the epoxy with the curing agent: *r* = 0.75 represents 25% less amine curing agent than that used in the stoichiometric mixing ratio; on the other hand, *r* = 1.25 represents 25% excess amine curing agent over epoxy resin. After pouring the mixture into the mold, a metal lid was placed on top, and the assembled mold was put into hot-press machine with applying force of 2 MPa to the mold. The curing time for every condition was set to 60 min. In addition, the effect of curing temperature was investigated. The mixture was cured at different specific curing temperatures of *T***_C_** = {90, 130, 170 and 210} °C. The mold was then kept under applying force until it cooled down to room temperature prior to taking out the specimens for further measurement. The total variable parameters in this study followed our previous work [14] and are summarized in Table 1. For the conditions of liquid DGEBA, the weight of the cured epoxies was set to be sufficient for the metal mold at 16 g, whereas the weight of the cured epoxies with the conditions of solid DGEBA was set at 24 g.

The single-lap shear specimens were prepared and tested for adhesive joint strength using the same procedure as described in our previous work. The epoxy resin and curing agent were preheated and then hand-mixed for a few seconds until achieving a homogeneous phase. Subsequently, the mixture was poured and spread onto an aluminum substrate (A6061P-T6, dimensions: 100 × 25 × 2 mm) over an area of 25 × 12.5 mm. The adhesive thickness was controlled by adding 0.1 parts per hundred resin of spherical glass beads (Fujiseisakujo, Tokyo, Japan). A second aluminum substrate was then placed on top of the adhesive overlapping the first substrate, creating a sandwich specimen. The specimens were clamped and subjected to the specific curing temperature in an oven for 60 min. Adhesive joint strength testing was conducted using a 10-kN AG-X plus series universal tensile testing machine (Shimadzu, Kyoto, Japan). To ensure data reliability, at least two specimens were fabricated for each measurement. The results are reported as the average value along with the standard deviation [14].

#### 2.1.3. Modulus Testing Technique

Shore hardness measurements of the specimens were performed using durometer hardness testing tools. After pressing the indentation on top of the specimen surface, the indenter of the durometer pierced the specimens, and then, the hardness values were observed by the gauge of the durometer. There are 2 types of durometer hardness testing tools: Shore A and Shore D, which were performed in this study. Shore A is appropriate for the soft materials that are in the range between 20A and 80A; on the other hand, Shore D is suitable for the hard materials that are in the range of 80A to 85D. After measuring hardness values, it was converted into S by the following Equation (1). Then, the elastic modulus was calculated by the relationship between hardness and elastic modulus via Equation (2) as follows [15]:

(1) $$S=\left\{\begin{array}{c} \;\;\;\;\;Shore\;A\;\;\;\;\;\;\;\;\;20A<S<80A\\ Shore\;D+50\;\;\;\;\;\;80A<S<85D \end{array}\right.$$

(2) $$logE_{0}=0.0235S-0.6403$$

The specimens were placed on the rigid surface prior to measuring the hardness at five points on the topside of the specimens in an effort to minimize variation. Every measurement point was at least 6.0 mm away from the other measuring points and at least 12.0 mm away from any edge of the specimens as illustrated in Figure S1b [17]. Areas of the specimens with trapped gas bubbles must be avoided when measuring the hardness. The elastic modulus of each point was independently calculated, and then, the average value with standard deviation was reported for each specimen.

### 2.2. Multi-Objective Optimization Approaches

#### 2.2.1. Dataset Preparation

The total number of possible conditions in this study was 256, which consisted of 4 *M*_wE_ times 4 *M*_wC_ times 4 amine-to-epoxide ratios times 4 curing temperatures (Table 1). However, the initial dataset for machine learning model training was 32 conditions according to our previous work [14]. According to our previous study, these 32 conditions of the initial dataset were selected by applying experimental techniques using four-by-four Graeco–Latin square design in order to uniformly distribute the experimental conditions [19,20]. There were two properties studied for each condition, which consisted of adhesive joint strength and elastic modulus. Datasets of adhesive joint strength were obtained from our previous work; on the other hand, datasets of elastic modulus were obtained by conducting experiments with the same conditions.

#### 2.2.2. Cross-Validation Technique

Machine learning modeling was carried out using the Python programming language. Several Python packages were applied in this work from the scikit-learn library, for example, data splitting, cross-validation, and machine learning model training and testing. An initial dataset of 32 conditions, each consisting of four variable parameters with two properties, was split into a training set and a testing set in order to train the machine learning model and evaluate its accuracy. In order to uniformly utilize all 32 conditions in the initial dataset as a training set and testing set, the K-fold cross-validation technique was introduced to split these initial datasets. K-fold cross-validation is a technique used for evaluating the performance of a model by dividing the dataset into multiple smaller folds. This method helps to reduce overfitting and provides a more accurate estimate of a model’s performance on unseen data. The initial dataset was randomly split into K equal-sized folds. In this study, the initial dataset was divided into K = 32 folds. Then, the machine learning model was trained on the data from the remaining K − 1 = 31 folds and evaluated for accuracy of models on the data from the remaining fold. This process was repeated K times, with each fold being used as the validation set only once. After that, the accuracy score of the machine learning models for each K times was calculated and then averaged to obtain the accuracy score of that model. The model with the highest accuracy score was chosen for further use. K-fold cross-validation is able to reduce the possibility of overfitting by training and evaluating the model on different folds of the data. This technique provides a more reliable estimate of the model’s performance on unseen data, as it reduces the impact of any single-fold peculiarities on the overall performance score [21].

#### 2.2.3. Modeling and Prediction

Seven machine learning algorithms were investigated in order to find the best model in this study. The Ridge regression algorithm is generally used to deal with a problem called multicollinearity. This problem occurs when the predictor variables—variables that are used to predict the outcome variable—in a regression model are highly correlated with each other. As a result, this can be difficult to estimate the effects of each predictor variable on the outcome variable accurately. The Ridge regression solves this problem by adding a small number to the diagonal of the matrix of predictor variables. By adding this number, the Ridge regression shrinks the estimates of the coefficients towards zero, which reduces their impact on the outcome variable. Thus, the Ridge regression algorithm is a technique that helps to improve the accuracy and reliability of estimates in regression models when there is multicollinearity among predictor variables [22]. Likewise, the Lasso regression algorithm is commonly used to prevent overfitting in linear regression models. However, the main difference between the Lasso and Ridge regression is the method used to prevent overfitting. Unlike the Ridge algorithm, the Lasso shrinks some of the coefficients to exactly 0, effectively performing variable selection and making the model simpler [23]. The Elastic net algorithm combines the strengths of both the Ridge and Lasso methods, while the Ridge regression does not perform variable selection and the Lasso can struggle with highly correlated predictors, to provide an improved approach for regularization and variable selection [24]. A new K-nearest neighbor (k-NN) algorithm utilizes the neighborhood points in the training sample. For each new data point, the algorithm finds the K nearest neighbors based on Euclidean distance. Then, the output variable for a new data point is predicted as the average of the output variables of its K nearest neighbors. This k-NN regression algorithm is a simple and intuitive algorithm that can work well for small datasets [25]. In cases of complex nonlinear relationships between the input and output variables, the Decision Tree Regression (DTR) algorithm can handle these cases efficiently. The DTR algorithm creates a tree-like structure where each internal node represents a decision based on a feature and threshold value, and each leaf node represents a prediction for the output variable. Then, the DTR algorithm selects the feature and threshold value that best split the data into two subsets that are as homogeneous as possible with respect to the output variable [26]. In order to improve the accuracy of the model, the Random Forest algorithm was introduced as an extension of the DTR algorithm. The principle of the Random Forest is to construct multiple decision trees at the training step and combine their predictions to make a final output variable prediction. Each tree is built using a random subset of the features and data points, which helps to reduce the correlation between the trees and improve the diversity of the forest. By combining the predictions of multiple trees, the Random Forest can capture more of the underlying patterns in the data and make more accurate predictions [27]. Lastly, the Gradient boosting algorithm was one of the candidate algorithms in this study. This algorithm works by iteratively adding decision trees to the model, with each tree attempting to correct the errors of the previous tree. The algorithm uses gradient descent optimization to minimize the loss function, which measures the difference between the predicted values and the actual values [28].

All seven machine learning algorithms were trained independently by using the initial dataset with the K-fold cross-validation technique. The accuracy of each algorithm was evaluated by the calculation of three tools: the coefficient of determination (R^2^ score), the mean absolute error (MAE) and the root-mean-square error (RMSE). The algorithms with a R^2^ score close to 1 show higher accuracy. On the other hand, the algorithms with a lower value of MAE and RMSE show higher accuracy. The calculation of these three tools utilized the prediction results from the testing dataset and the measured results from the experiment. Moreover, the predictions of both adhesive joint strength and elastic modulus were carried out simultaneously in this step. 

The model with the highest accuracy was selected to perform further prediction of the total 256 conditions. Furthermore, the K-fold cross-validation technique was also applied in this step in order to average the prediction results of each fold in each condition. Hence, the relationship between adhesive joint strength and elastic modulus for each condition was observed. In addition, the standard deviation of each prediction result was obtained. 

Three predicted results with high deviations from three regions: low adhesive joint strength with low elastic modulus, low adhesive joint strength with high elastic modulus and high adhesive joint strength with high elastic modulus. These were selected to perform an active learning approach. The experimental conditions at these three selected points were conducted for the experiment. Then, the additional dataset (*m_i_* = 3) of the measured adhesive joint strength and elastic modulus was added to the initial dataset (*n_i_* = 32) to make a new dataset (*n* = *n_i_* + *m_i_* = 32 + 3 = 35) for the second active learning cycle, and so on. The overall process started with machine learning model training once again in order to improve the model accuracy as well as the standard deviation of the predicted results; this process is called the active learning approach. This active learning approach was repeated until the average values of the prediction errors (the standard deviation of predicted results) were comparable to the experimental errors; then, the active learning loop was terminated. 

After termination of the active learning loop, machine learning model accuracy and the standard deviation of the predicted results were collected. In addition, the correlation between adhesive joint strength and elastic modulus properties was observed by plotting the predicted 256 conditions from the last active learning cycle. Moreover, the trade-off line of these two properties, represented by the Pareto frontier line, was drawn by connecting the boundary points. These data were kept for investigation in the next step.

#### 2.2.4. Bayesian Optimization

**Figure 2:**
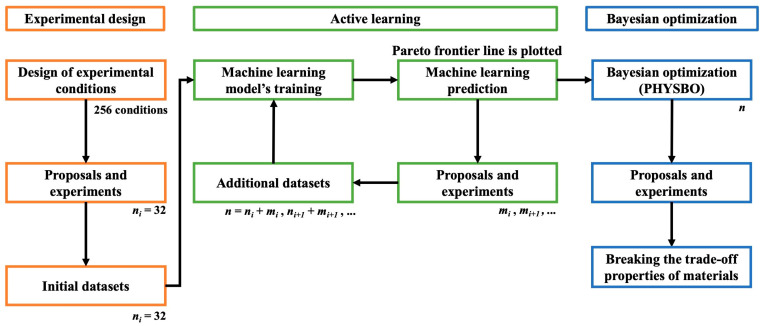
Illustration of the overall workflow, consisting of three stages: (i) experimental design, (ii) active learning cycle and (iii) Bayesian optimization, where *n* refers to the total number of datasets, *i* refers to the first cycle and *m* refers to the number of additional datasets.

In this study, Bayesian optimization was performed using PHYSBO [29], a Python library for Bayesian optimization, in order to search for extended conditions outside the Pareto frontier line. In this step, the variable parameters of amine-to-epoxide ratios together with curing temperatures were studied as a continuous value. The amine-to-epoxide ratios ranged from 0.75 to 1.50 with 0.01 increment steps. On the other hand, the curing temperatures ranged from 90 °C to 210 °C with an increment step of 1 °C. Nevertheless, the *M*_wE_ and *M*_wC_ parameters were maintained as discrete values because of the limitations in the supply of these commercially available materials. The summary of variable parameters for Bayesian optimization is shown in Table 2. All of measured data was utilized as an input dataset (*n*) in the PHYSBO with the adjusted settings for a multi-objective optimization case with two objective numbers. Additionally, the Thompson sampling method [30] was selected to search for conditions beyond the Pareto frontier line from the active learning stage. In the end, the conditions proposed by PHYSBO were conducted the experiments to confirm the results. The pipeline of the overall workflow is illustrated in Figure 2.

## 3. Results

### 3.1. Experiments

**Figure 3:**
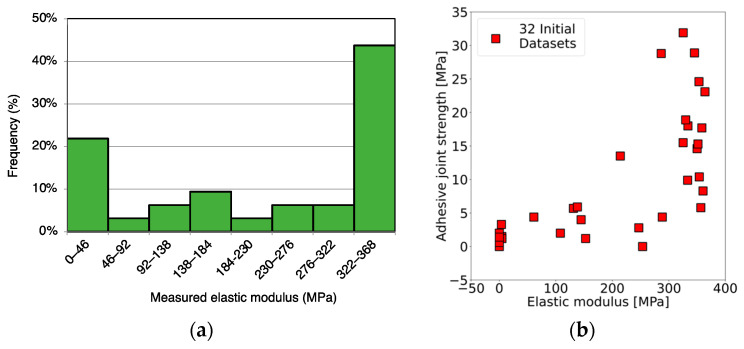
Experimental results of adhesive joint strength (MPa) and elastic modulus (MPa); (**a**) distribution chart of elastic modulus from 32 conditions of the initial dataset, (**b**) characteristics of adhesive joint strength (MPa) and elastic modulus (MPa) properties on 32 conditions.

The polymeric specimens were fabricated successfully, ensuring smooth surfaces and uniformity. Eye inspection revealed the absence of voids and bubbles within the specimens. The example of specimens is illustrated in the Figure S2. The specimens were then measured for the hardness. The elastic modulus was calculated after measuring the hardness of each specimen. The highest elastic modulus was observed at 363.9 MPa, whereas the lowest elastic modulus was observed at 0 MPa because of the incompletely cured specimen. All of the experimental results with variable parameters are listed in Table S1 (Supplementary Materials). The distribution of elastic modulus was plotted as the percentage of the total 32 specimens within specific ranges of elastic modulus values to examine the distribution of the dataset. For example, 22% indicates that 7 out of 32 specimens have elastic modulus values in the range of 0 to 46 MPa. This distribution demonstrated a well-spread dataset, as illustrated in Figure 3a. Together with the adhesive joint strength results from previous work [14], these two properties were observed as the characteristics in Figure 3b. 

### 3.2. Multi-Objective Optimization

#### 3.2.1. Machine Learning Model Selection and Model Training

The accuracy of seven machine learning algorithms was evaluated by applying the 32 initial datasets as a training and testing set along with the K-fold cross-validation technique. Three evaluation tools, R^2^ score, MAE and RMSE, were used to check for accuracy, and the results are reported in Table 3. The Ridge, Lasso and Elastic net algorithms manifested a similar level of accuracy. As a result, the fundamental structure of these three algorithms is a linear regression model; thus, they assumed a linear relationship between the input features and the target variables. Even though the regularization techniques of these three models are different, the accuracies are close to each other. Therefore, the initial dataset showed a non-linear relationship. The k-NN algorithm provided a slightly lower accuracy compared to the linear regression models because the k-NN model makes predictions based on the similarity of data points in the input space without assuming a specific functional form for the underlying relationship. Therefore, these four models were not selected for the reason that they are inappropriate algorithms for the dataset and provide low accuracy. The DTR model reported the most obvious lowest accuracy among the others; for this reason, it was discarded. The Random Forest and Gradient boosting models showed high accuracy on both adhesive joint strength and elastic modulus properties; however, the Gradient boosting model could achieve higher accuracy in adhesive joint strength properties. Although the Random Forest and Gradient boosting algorithm are both ensemble learning techniques, their approaches to building and combining individual models are different. Random Forest creates diverse and independent trees in parallel, while Gradient boosting builds trees sequentially, focusing on correcting errors made by the ensemble. Considering its better performance, the Gradient boosting model was nominated for further prediction.

#### 3.2.2. Machine Learning Prediction and Proposals for Experiments

**Figure 4:**
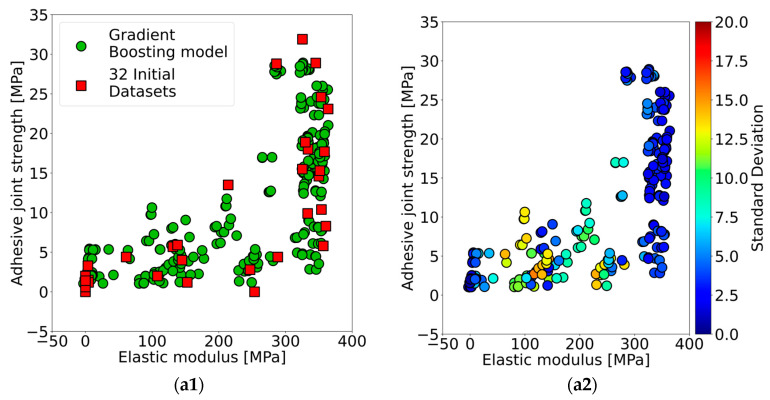

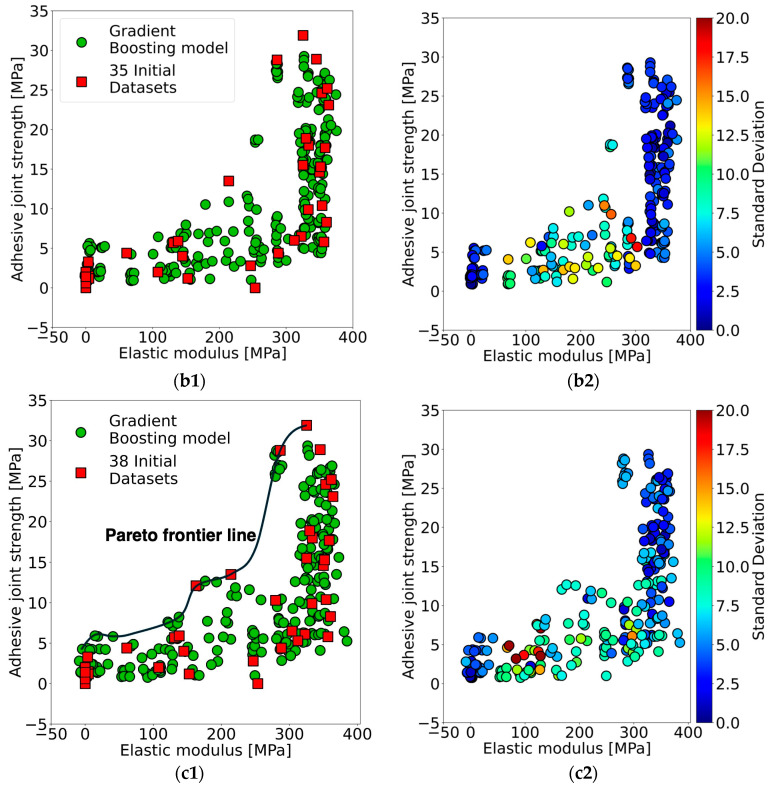
Prediction of the adhesive joint strength (MPa) and elastic modulus (MPa) on 256 conditions compared with initial datasets after (**a1**) active learning cycle 1; *n_i_* = 32, (**b1**) cycle 2; *n* = 35, and (**c1**) cycle 3; *n* = 38, as well as the averaged standard deviation of each predicted result (**a2**) cycle 1, (**b2**) cycle 2 and (**c2**) cycle 3.

The Gradient boosting model was trained by using an initial dataset of 32 conditions as well as applying the K-fold cross-validation technique. Firstly, the averaged prediction on both adhesive joint strength and elastic modulus of a testing dataset from each fold was reported by comparing it with measured results from the experiment as plotted in Figure S3. The diagonal dash line refers to the same value between the prediction and experimental results. Secondly, the averaged prediction results of adhesive joint strength and elastic modulus for the 256 possible conditions, consisting of four values of the four variable parameters as shown in Table 1, were conducted and reported together with the initial dataset as shown in Figure 4(a1–c1). Lastly, the standard deviation of predicted adhesive joint strength and elastic modulus at each prediction point was averaged to be reported as a representative of deviation value, and it is shown in the color scale in Figure 4(a2–c2).

In the first cycle (Figure S3a), the prediction of the testing dataset showed a high deviation at the high adhesive joint strength of above 10 MPa; on the other hand, a high deviation could be observed at the middle range of elastic modulus from 100 to 350 MPa. In addition, the prediction of a total of 256 possible conditions on both adhesive joint strength and elastic modulus was successfully carried out as shown in Figure 4(a1). The prediction results showed good distribution along with the initial dataset (*n_i_* = 32); however, prediction could not be observed in the area of high adhesive joint strength with low elastic modulus (top-left zone). The missing data indicate the possibility of a trade-off characteristic between these two properties. In the Figure 4(a2), the standard deviation of prediction results from the first active learning cycle showed the highest deviation at 15.5 MPa. Moreover, regarding the percentage of high-deviated prediction results, higher than half of the highest deviation, was observed at 9%. After that, the high-deviation conditions from three regions—low–low, low–high and high–high adhesive joint strength and elastic modulus, respectively—were selected for further experiments as listed in Table 4 for condition numbers 33, 34 and 35. The experimental results from the proposed conditions indicated almost similar results to the predictions except for the elastic modulus of condition number 35. This is because high deviation leads to low accuracy in the prediction.

**Figure 5:**
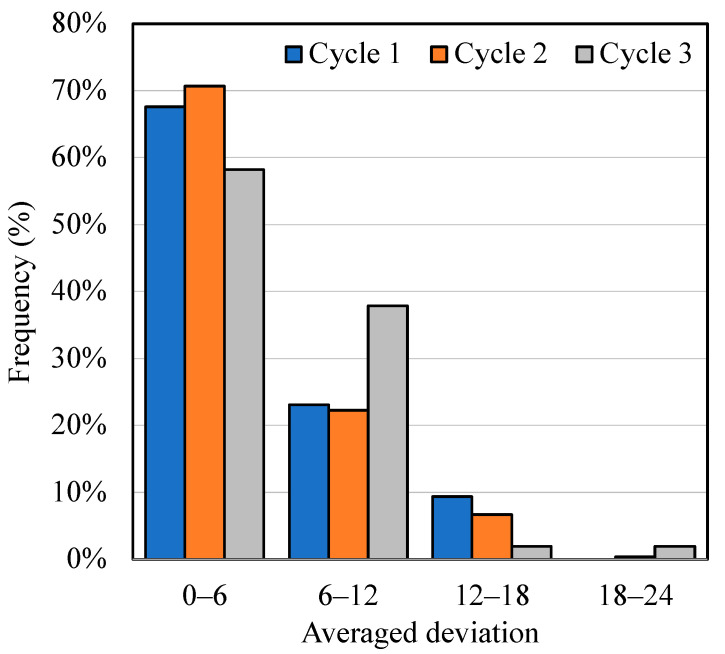
Distribution of averaged deviation from active learning cycle 1, cycle 2 and cycle 3.

The measured results of both adhesive joint strength and elastic modulus from the experiment according to conditions 33, 34 and 35 were added to the initial dataset for the second cycle of active learning. These new initial datasets (*n* = 32 + 3 = 35) were utilized to train the machine learning model of Gradient boosting once again. Accompanying the K-fold cross-validation technique, the predictions of a testing set were compared to the measured testing set itself, and they were plotted as shown in Figure S3b. The predicted adhesive joint strength still performed with a high deviation at the high predicted values which were the same as those of the first cycle. In contrast, the model accuracy could be improved by observing a better R^2^ score, MAE and RMSE after applying this active learning approach. On the other hand, the elastic modulus properties not only showed high-deviation prediction results in the middle range but also obtained lower accuracy on the R^2^ score, MAE and RMSE. In Figure 4(b1), the prediction of 256 conditions was reported together with a new initial dataset (*n* = 35) in this second cycle. The absence of the predicted results could still be discovered in the area of low elastic modulus with high adhesive joint strength. However, the predictions were very well distributed along with the initial dataset. In the deviation point of view, the averaged standard deviation between adhesive joint strength and elastic modulus at 18.1 MPa was observed to have the highest values as shown in Figure 4(b2). Moreover, the predicted results at the area of high elastic modulus performed with an outstandingly low deviation according to the prediction of the testing dataset from Figure S3b. Nevertheless, comparing the percentage of high-deviation prediction results between the first cycle and the second cycle, it could be found that there was a 2% improvement in decreasing the high-deviation prediction results as shown in Figure 5. After that, a new set of three conditions with high deviation, as listed in Table 4 for the conditions 36, 37 and 38, were chosen again in order to conduct an experiment for a further active learning cycle. Additionally, the measured results from the experiment are reported in Table 4. The experimental results indicated an improvement in the deviation compared to the predictions. Subsequently, these three results were appended to the initial dataset for the third active learning cycle.

The total 38 conditions (*n* = 35 + 3 = 38) were utilized as an initial dataset in this third active learning loop. The process was repeated by training the Gradient boosting model along with applying the K-fold cross-validation technique, then predicting the testing set and evaluating the model accuracy. The third cycle prediction of the testing set is reported in Figure S3c together with the model accuracies of both adhesive joint strength and elastic modulus. The model accuracy of the adhesive joint strength slightly decreased from the second cycle; however, it could exhibit better performance compared to the first cycle. On the other hand, the accuracy of the elastic modulus kept decreasing from the first cycle. In spite of that, it is obvious that the prediction of a testing set deviated from the diagonal dash line much less compared to that of the first and second cycles. Then, the prediction of 256 conditions was carried out and reported together with an initial dataset of 38 conditions as shown in Figure 4(c1). The same tendency as seen in the previous cycle was observed: the predictions could not be found in the area of high adhesive joint strength with low elastic modulus. Therefore, this could imply a trade-off between these two properties after three cycles of the active learning loop. Additionally, the Pareto frontier line was drawn to represent a trade-off boundary connecting all of the results at the top-left edge. In Figure 4(c2), the maximum deviation of these two properties is 21.0 MPa, and the extremely high-deviation results show less than 10 conditions. Moreover, the deviation of the overall predicted results improved because of the clearly seen shift in color scale from yellow in the first cycle to light blue in this cycle. Furthermore, a significant enhancement in prediction accuracy was observed, with a 50% reduction in high-deviation outcomes from the first to the third cycle, as illustrated in Figure 5. The active learning cycle terminated once the average standard deviation of predictions closely matched that of experimental results for both adhesive joint strength and elastic modulus. Thus, the errors in the predictions were acceptable according to the errors from the experiments. In conclusion, the active learning approach was able to balance machine learning model accuracy and the deviation of each single predicted result. Consequently, the Pareto frontier line could be obtained from the predictions of 256 conditions (Figure 4(c1)), and it was highly reliable after achieving three cycles of the active learning loop. 

The influence of each variable parameter on the prediction of both properties is illustrated in Figure S4. It was observed that the molecular weight of the epoxy resin (*M*_wE_) exhibited minimal impact on both properties as indicated by its ability to perform consistently across a wide range of predicted adhesive joint strength and elastic modulus (Figure S4a). However, the epoxy resin with molecular weight of 370 g/mol offers significant advantages in processability due to its liquid phase state. In contrast, the molecular weight of the curing agent (*M*_wC_) demonstrated a significant impact on the predicted properties. Lower-viscosity curing agents with *M*_wC_ values of 230 and 400 g/mol resulted in a prediction of high adhesive joint strength and elastic modulus. Conversely, higher-viscosity curing agents with *M*_wC_ values of 2000 and 4000 g/mol only achieved a maximum adhesive joint strength of 11.8 MPa. Regarding elastic modulus, predictions with a modulus below 300 MPa were challenging to obtain using curing agents with an *M*_wC_ of 230 g/mol, suggesting the necessity for higher molecular weight curing agents for low modulus adhesives. Moreover, the control of predicted adhesive joint strength and elastic modulus properties is more readily achieved through the molecular weight of the curing agent (Figure S4b). The impact of the amine-to-epoxide ratio is shown in Figure S4c, indicating its uniform distribution across the range of predicted properties. Higher ratios notably enhanced adhesive joint strength, particularly surpassing 16 MPa, while elastic modulus values between 100 and 200 MPa were predominantly attained with a ratio of 0.75. Additionally, it was observed that curing temperatures at 90 °C resulted in predicted adhesive joint strengths below 12 MPa. Conversely, curing temperatures exceeding 90 °C facilitated a more favorable distribution for both predicted adhesive joint strength and elastic modulus properties, as illustrated in Figure S4d.

#### 3.2.3. Bayesian Optimization

**Figure 6:**
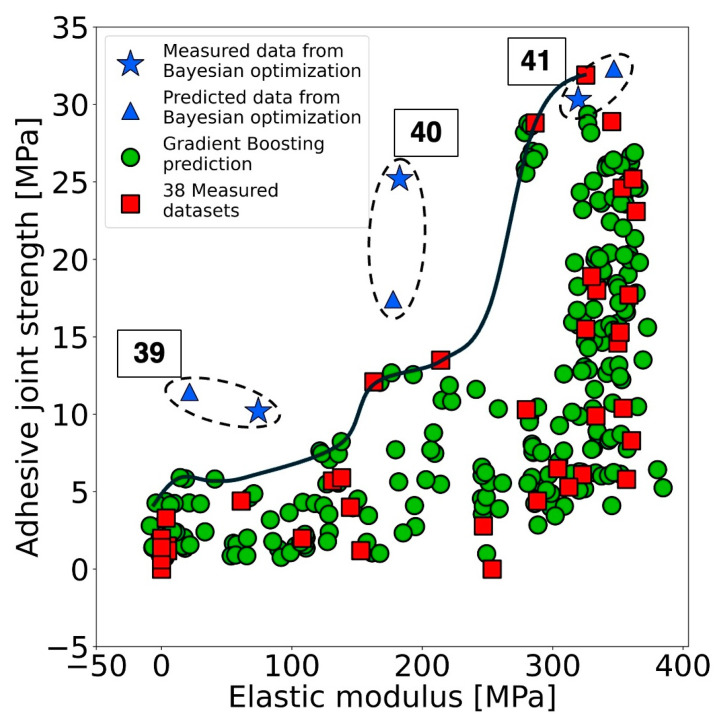
The experimental results and proposed conditions for adhesive joint strength (MPa) and elastic modulus (MPa) from multi-objective optimization using PHYSBO along with the predictions from active learning and a Pareto frontier line.

According to the trade-off behavior proposed in the previous section (Figure 4(c1)), adhesive epoxy materials exhibiting high adhesive joint strength but low elastic modulus have yet to be developed through the machine learning approach. However, such a formulation, which shows high adhesive joint strength and low elastic modulus, could offer considerable advantages, particularly in applications requiring efficient force absorption in adhesive materials. To address this, a new initial dataset comprising 38 conditions was utilized for further investigation, employing Bayesian optimization to identify conditions conducive to achieving an adhesive epoxy with the desired properties. In this stage, PHYSBO which is a Python library for Bayesian optimization studies focusing on the physics, chemistry and materials science fields was performed to optimize this multi-objective problem. Four variable parameters with discrete and continuous types, as summarized in Table 2, were studied. The proposed conditions from Bayesian optimization were separately reported in four figures, as shown in Figures S5–S8. The predicted adhesive joint strength and elastic modulus from Bayesian optimization were plotted individually by varying the continuous parameters of amine-to-epoxide ratio and curing temperature; however, the predictions by varying discrete parameters of *M*_wE_ and *M*_wC_ were plotted one by one in separate charts. The predicted adhesive joint strength and elastic modulus were mainly changed by varying *M*_wE_ and *M*_wC_. Particularly, changing the M_wC_ along its four values could obtain a variety of predicted adhesive joint strength and elastic modulus results. This was confirmed by the influence of *M*_wC_ on prediction of both properties after the final active learning cycle (Figure S4). On the other hand, the influence of amine-to-epoxide ratio and curing temperature exhibited a notable influence on the predicted results, particularly noticeable at lower values of *M*_wE_ and *M*_wC_. Afterward, the conditions proposed by Bayesian optimization, where the predicted results exceeded the trade-off boundary, were selected to overcome the Pareto frontier line. Considering the discussion from the previous section regarding the influence of each variable parameter, three conditions with predicted results beyond the Pareto frontier line were selected as listed in Table 5. Additionally, the experimental results corresponding to these conditions were reported in Table 5 and plotted alongside the Pareto frontier line in Figure 6. Notably, conditions 39 and 40 exhibited adhesive joint strengths of 10.2 MPa and 25.2 MPa, respectively, along with elastic modulus of 74.2 MPa and 182.5 MPa, positioning them further from the trade-off limit. Furthermore, a specimen with desirable properties as of high adhesive joint strength together with low elastic modulus was successfully fabricated as polymeric specimen number 40. A comparison of the appearance between specimen number 40 and the specimen with high adhesive joint strength and elastic modulus (number 26) was conducted to confirm the difference, as reported in Figure S9. Significantly, specimen number 40 displayed superior ductile characteristics compared to specimen number 26, despite both exhibiting the same level of adhesive joint strength values. Additionally, elasticity behavior was demonstrated, as illustrated in Figure S10. Specimen number 26, with a higher elastic modulus, retained its shape when subjected to a 100 g load, whereas specimen number 40, with a lower elastic modulus, exhibited deformation under the same load. Therefore, not only was the trade-off between adhesive joint strength and elastic modulus optimized, but also, a specimen with the desired properties was successfully fabricated by adhering to the suggested conditions derived from the Bayesian optimization approach.

## 4. Conclusions

Multi-objective optimization was employed to analyze the adhesive joint strength and elastic modulus properties of commercially available epoxy resin (DGEBA) with an amine-terminated curing agent (Jeffaime™). Four variable parameters, consisting of molecular weight of DGEBA (*M*_wE_), molecular weight of Jeffamine™ (*M*_wC_), amine-to-epoxide ratio (*r*) and curing temperature (*T*_C_), were investigated. In this study, the elastic modulus was measured using a specially designed metal mold apparatus. The polymeric specimens were fabricated with smooth surfaces and homogeneity. The elastic modulus values were derived from hardness measurements of each specimen. Subsequently, these values, along with the adhesive joint strength values from a previous study, constituted the initial dataset of 32 conditions to train the machine learning model. Among the seven machine learning algorithms evaluated, the Gradient Boosting model exhibited the highest accuracy and was selected for further analysis. The initial dataset was then utilized in an active learning approach to train the Gradient Boosting model and make predictions, resulting in a 50% reduction in deviation of predicted results and improved balance in predictive accuracy across adhesive joint strength and elastic modulus properties after the third cycle of the active learning approach. Consequently, the Pareto frontier line showing the trade-off boundary between these two properties was able to be reliably presented. The missing prediction area at high adhesive joint strength with low elastic modulus was observed as the trade-off area. The influence of each variable parameter on both adhesive joint strength and elastic modulus properties was examined. The results indicated that an epoxy resin with *M*_wE_ of 370 g/mol offered optimal processability because of its liquid phase state, the *M*_wC_ of the Jeffamine™ curing agent played a crucial role in controlling properties to achieve specimens with high adhesive joint strength and low elastic modulus, an amine-to-epoxide ratio (*r*) of 0.75 was suitable for fabricating adhesive epoxy with lower elastic modulus, and a curing temperature (*T*_C_) above 90 °C was necessary to maintain adhesive ability.

Bayesian optimization was employed to address the trade-off challenges. Utilizing a dataset of *n* = 38 (initial 32 datasets plus an additional 6), PHYSBO was employed to optimize the adhesive joint strength and elastic modulus properties of the adhesive epoxy system. Molecular weights of DGEBA (*M*_wE_) and Jeffamine™ (*M*_wC)_ were treated as discrete variables, while amine-to-epoxide ratio (*r*) and curing temperature (*T*_C_) were explored as continuous variables. The PHYSBO approach effectively predicted and suggested properties for this adhesive epoxy system across a vast array of conditions, up to 147,136 in total. Upon experimentation, the optimized conditions were validated, successfully transcending the trade-off boundary (Pareto frontier line) with adhesive joint strength reaching 25.2 MPa and elastic modulus at 182.5 MPa, respectively. The fabricated specimen exhibited superior elastic behavior. This multi-objective optimization strategy provides valuable insights into the curing conditions for the studied adhesive epoxy system, elucidating the impact of each variable parameter on mechanical properties. Moreover, Bayesian optimization demonstrates its capability to efficiently suggest conditions for desired properties, accelerating the overall process, reducing costs, and time consumption, while also enabling the breakthrough of trade-off constraints in the adhesive epoxy system.

## Figures and Tables

**Table 1:** Summary of variable parameters for experiments [14].

Epoxy Resin		Curing Agent		Amine to Epoxide Ratio (*r*)	*T*_C_ (°C)
*M*_wE_ (g/mol)	Appearance	*M*_wC_ (g/mol)	Appearance		
370	Liquid	230	Viscous	0.75	90
1650	Solid	400	Viscous	1.00	130
2900	Solid	2000	Highly viscous	1.25	170
3800	Solid	4000	Highly viscous	1.50	230

**Table 2:** Summary of variable parameters for Bayesian optimization.

Parameters	Values				Step	Type
*M*_wE_ (g/mol)	370	1650	2900	3800	-	Discrete
*M*_wC_ (g/mol)	230	400	2000	4000	-	Discrete
*r*	0.75–1.50				0.01	Continuous
*T***_C_** (°C)	90–120				1	Continuous

**Table 3:** Comparison of the accuracy of seven machine learning models represented by R^2^ score, MAE and RMSE.

		Ridge	Lasso	Elastic Net	k-NN	DTR	Random Forest	Gradient Boosting
Adhesive joint strength	R^2^ score	0.42	0.43	0.42	0.40	0.18	0.51	0.60
	MAE	5.7	5.6	5.7	5.4	5.7	4.7	4.3
	RMSE	7.2	7.1	7.2	7.3	8.5	6.6	6.0
Elastic modulus	R^2^ score	0.57	0.58	0.58	0.54	0.76	0.82	0.82
	MAE	70.5	69.3	70.8	70.8	43.9	41.9	40.0
	RMSE	91.7	91.3	91.5	95.3	68.5	60.2	59.5

**Table 4:** Prediction of adhesive joint strength (MPa) and elastic modulus (MPa) of the proposed conditions and the experimental results.

From Cycle	No.	Variable Parameters				Predicted Adhesive Joint Strength (MPa)	Predicted Elastic Modulus (MPa)	Measured Adhesive Joint Strength (MPa)	Measured Elastic Modulus (MPa)
		*M*_wE_ (g/mol)	*M*_wC_ (g/mol)	*r*	*T*_C_ (°C)				
1	33	370	230	1.00	130	28.1 ± 2.5	335.8 ± 8.5	25.2 ± 2.3	361.5 ± 5.2
	34	1650	400	1.25	90	6.8 ± 0.7	314.4 ± 8.0	6.1 ± 1.8	322.7 ± 8.8
	35	2900	2000	1.50	170	6.4 ± 0.8	95.3 ± 24.4	5.3 ± 2.7	312.3 ± 14.9
2	36	370	400	0.75	130	18.4 ± 1.3	253.7 ± 18.0	12.1 ± 1.8	162.8 ± 11.8
	37	2900	2000	1.50	210	7.5 ± 0.8	291.9 ± 36.0	10.3 ± 2.9	279.7 ± 20.1
	38	3800	2000	1.50	90	6.4 ± 1.2	108.3 ± 25.0	6.5 ± 2.1	303.8 ± 18.3

**Table 5:** Proposed conditions from the Bayesian optimization step, and the experimental results according to the conditions.

No.	Variable Parameters				Predicted Adhesive Joint Strength (MPa)	Predicted Elastic Modulus (MPa)	Measured Adhesive Joint Strength (MPa)	Measured Elastic Modulus (MPa)
	*M*_wE_ (g/mol)	*M*_wC_ (g/mol)	*r*	*T*_C_ (°C)				
39	370	2000	1.46	129	11.5	21.5	10.2 ± 0.6	74.2 ± 3.3
40	370	400	0.75	168	17.4	177.5	25.2 ± 1.3	182.5 ± 7.9
41	370	230	1.13	160	32.3	346.8	30.3 ± 0.9	319.5 ± 17.4

## Data Availability

Data are contained within the article and Supplementary Materials.

## Supplementary Materials

The following supporting information can be downloaded at: https://www.mdpi.com/article/10.3390/ma17122866/s1, Figure S1: An illustration of (a) a metal mold according to French standard NFT76-142, (b) dimension of the specimens and the measuring points according to ASTM-D2240; Figure S2: An example of the specimen’s appearance with conditions 26 and 31; Figure S3: The averaged prediction of adhesive joint strength and elastic modulus from 32-fold cross-validation compared to its measured results from experiments as well as the accuracies of each active learning cycle; (a) 1st cycle, (b) 2nd cycle and (c) 3rd cycle; Figure S4: The influence of each variable parameter on the prediction of 256 conditions from the 3rd active learning cycle: (a) molecular weight of epoxy resin, (b) molecular weight of curing agent, (c) amine to epoxide ratio and (d) curing temperature; Figure S5: The proposed conditions from Bayesian optimization for adhesive joint strength (MPa) and elastic modulus (MPa) by varying three variable parameters—molecular weight of Jeffamine™ (g/mol), amine-to-epoxide ratio and curing temperature (°C)—with the molecular weight of DGEBA at 370 g/mol; Figure S6: The proposed conditions from Bayesian optimization for adhesive joint strength (MPa) and elastic modulus (MPa) by varying three variable parameters—molecular weight of Jeffamine™ (g/mol), amine-to-epoxide ratio and curing temperature (°C)—with the molecular weight of DGEBA at 1650 g/mol; Figure S7: The proposed conditions from Bayesian optimization for adhesive joint strength (MPa) and elastic modulus (MPa) by varying three variable parameters—molecular weight of Jeffamine™ (g/mol), amine-to-epoxide ratio and curing temperature (°C)—with the molecular weight of DGEBA at 2900 g/mol; Figure S8: The proposed conditions from Bayesian optimization for adhesive joint strength (MPa) and elastic modulus (MPa) by varying three variable parameters—molecular weight of Jeffamine™ (g/mol), amine-to-epoxide ratio and curing temperature (°C)—with the molecular weight of DGEBA at 3800 g/mol; Figure S9: The appearance of specimens after tearing by tensile force of the specimen with condition number 26 (high elastic modulus) shows less ductile behavior compared to the specimen with condition number 40 (less elastic modulus); Figure S10: Demonstration of the elasticity behavior of the specimens: (a) high elastic modulus specimen no. 26 before adding load, (b) after adding 100 g load, (c) low elastic modulus specimen no. 40 before adding load and (d) after adding 100 g load; Figure S11: A picture of actual metal mold consisting of a metal lid, Teflon tape, metal box, silicon rubber frame and adhesive epoxy specimen; Table S1: Experimental results of adhesive joint strength (MPa) and elastic modulus (MPa) of each condition consist of four variable parameters: molecular weight of DGEBA *M*_wE_ (g/mol), molecular weight of Jeffaime™ curing agent *M*_wC_ (g/mol), amine-to-epoxide ratio; *r*, and curing temperature *T*_C_ (°C). Initial dataset size *n_i_* = 32 samples. Refs [14,17] are cited in the supplementary materials.
