# Supplementary material for: Multi-Objective Optimization of Adhesive Joint Strength and Elastic Modulus of Adhesive Epoxy with Active Learning
Source: Materials (Basel). 2024 Jun 12;17(12):2866. doi: 10.3390/ma17122866 (PMC11204689; doi:10.3390/ma17122866)
Supplement: Supplementary file 1 [file materials-17-02866-s001.zip › materials-2996338-supplementary.pdf]

Supplementary information

For

Multi-Objective Optimization of Adhesive Joint Strength and Elastic Modulus of Adhesive Epoxy with Active Learning

Paripat Kraisornkachit [1,2], Masanobu Naito [1,2,*], Chao Kang [3] and Chiaki Sato [3]

[1] Data-Driven Polymer Design Group, Research Center for Macromolecules and Biomaterials, National Institute for Materials Science (NIMS), Ibaraki 305-0047, Japan
[2] Program in Materials Science and Engineering, Graduate School of Pure and Applied Sciences, University of Tsukuba, Ibaraki 305-8577, Japan
[3] Institute of Innovative Research (IIR), Tokyo Institute of Technology, Kanagawa 226-8503, Japan
* Correspondence: naito.masanobu@nims.go.jp

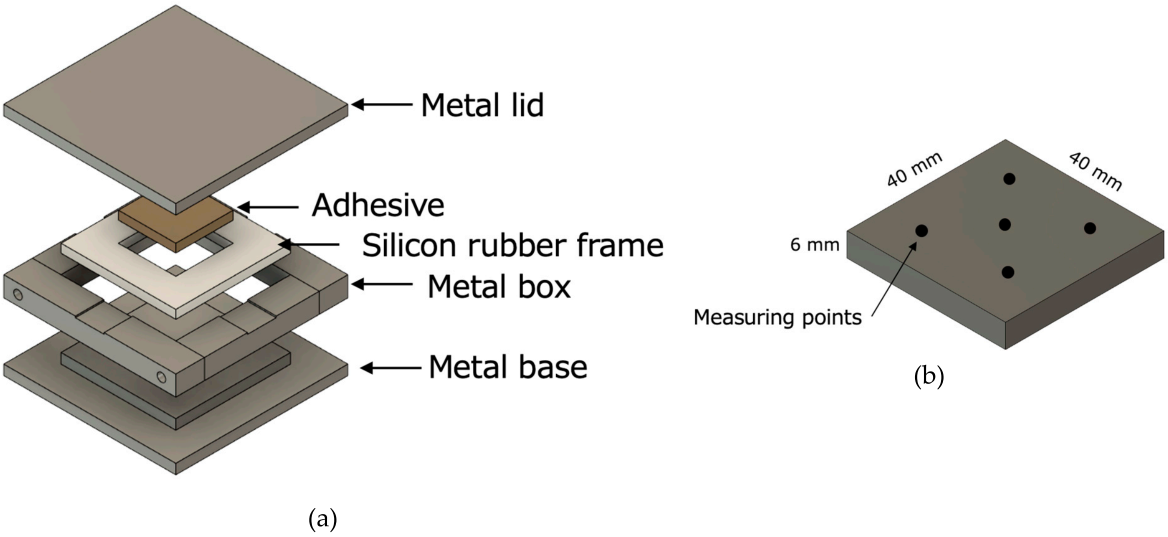

Figure S1. An illustration of (a) a metal mold according to French standard NFT76-142, (b) dimension of the specimens and the measuring points according to ASTM-D2240 [1].

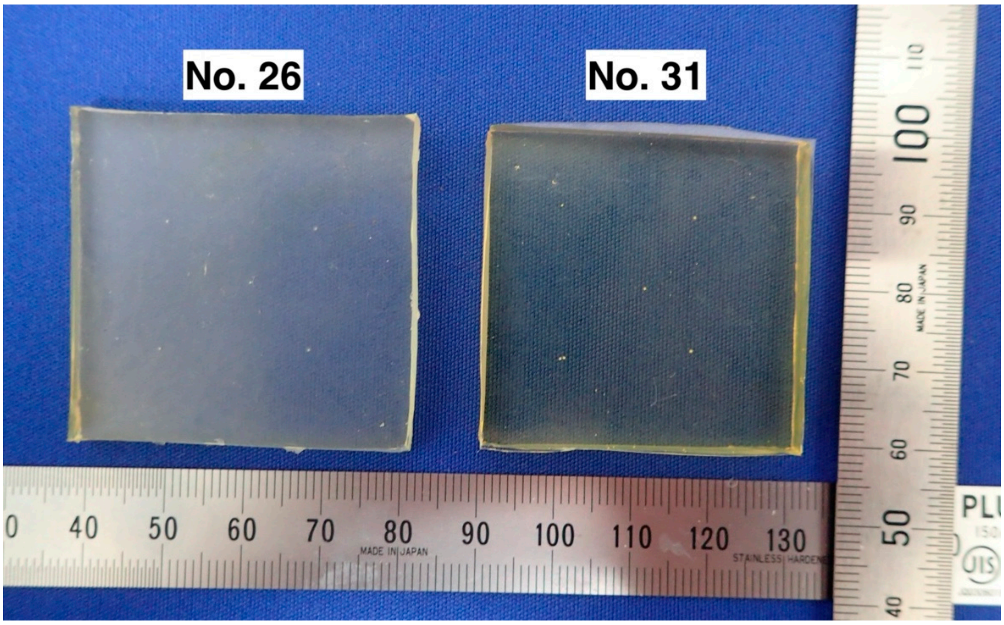

Figure S2. An example of the specimen's appearance with conditions 26 and 31.

Table S1. Experimental results of adhesive joint strength (MPa) [2] and elastic modulus (MPa) of each condition consist of four variable parameters; molecular weight of DGEBA Mw_E (g/mol), molecular weight of Jeffaime™ curing agent Mw_C (g/mol), amine-to-epoxide ratio; r, and curing temperature T_C (°C). Initial dataset size n_i = 32 samples.

No.	Variable parameters				Measured adhesive joint strength (MPa) [2]	Measured elastic modulus (MPa)
	Mw_E (g/mol)	Mw_C (g/mol)	r	T_C (°C)		
1	370	230	0.75	90	8.3 ± 1.8	360.3 ± 8.1
2	370	400	1.00	170	28.8 ± 0.7	286.3 ± 14.0
3	370	2000	1.25	210	1.5 ± 0.1	4.3 ± 0.1
4	370	4000	1.50	130	0.0 ± 0.0	0.0 ± 0.0
5	1650	230	1.00	130	18.0 ± 0.3	333.6 ± 18.3
6	1650	400	0.75	210	14.6 ± 1.0	349.9 ± 4.8
7	1650	2000	1.50	170	3.3 ± 0.7	3.6 ± 0.2
8	1650	4000	1.25	90	2.0 ± 0.1	0.0 ± 0.0
9	2900	230	1.25	170	17.7 ± 0.3	358.3 ± 1.7
10	2900	400	1.50	90	5.8 ± 0.5	356.5 ± 7.3
11	2900	2000	0.75	130	5.7 ± 0.3	131.0 ± 15.2
12	2900	4000	1.00	210	4.4 ± 0.5	288.2 ± 21.6
13	3800	230	1.50	210	15.3 ± 2.5	351.5 ± 7.4
14	3800	400	1.25	130	10.4 ± 0.1	353.8 ± 10.4
15	3800	2000	1.00	90	1.2 ± 0.0	152.7 ± 19.0
16	3800	4000	0.75	170	4.0 ± 0.1	144.7 ± 16.8
17	370	230	1.50	130	31.9 ± 0.1	325.3 ± 12.2
18	370	400	0.75	90	2.8 ± 1.5	246.8 ± 6.4
19	370	2000	1.00	170	1.2 ± 0.2	4.6 ± 0.1
20	370	4000	1.25	210	0.6 ± 0.0	0.0 ± 0.0
21	1650	230	1.25	90	9.9 ± 0.7	333.1 ± 21.9
22	1650	400	1.00	130	18.9 ± 0.8	329.8 ± 24.6
23	1650	2000	0.75	210	5.9 ± 0.7	138.1 ± 16.5
24	1650	4000	1.50	170	1.4 ± 0.5	0.0 ± 0.0
25	2900	230	1.00	210	23.1 ± 1.4	363.9 ± 7.0
26	2900	400	1.25	170	24.6 ± 0.5	353.2 ± 15.5
27	2900	2000	1.50	90	4.4 ± 0.1	61.1 ± 8.1
28	2900	4000	0.75	130	2.0 ± 0.6	107.9 ± 12.3
29	3800	230	0.75	170	15.5 ± 0.2	325.2 ± 9.3
30	3800	400	1.50	210	28.9 ± 0.6	345.3 ± 14.4
31	3800	2000	1.25	130	13.5 ± 0.7	214.0 ± 22.0
32	3800	4000	1.00	90	0.0 ± 0.0	253.5 ± 22.2

(a) 1st cycle of active learning loop

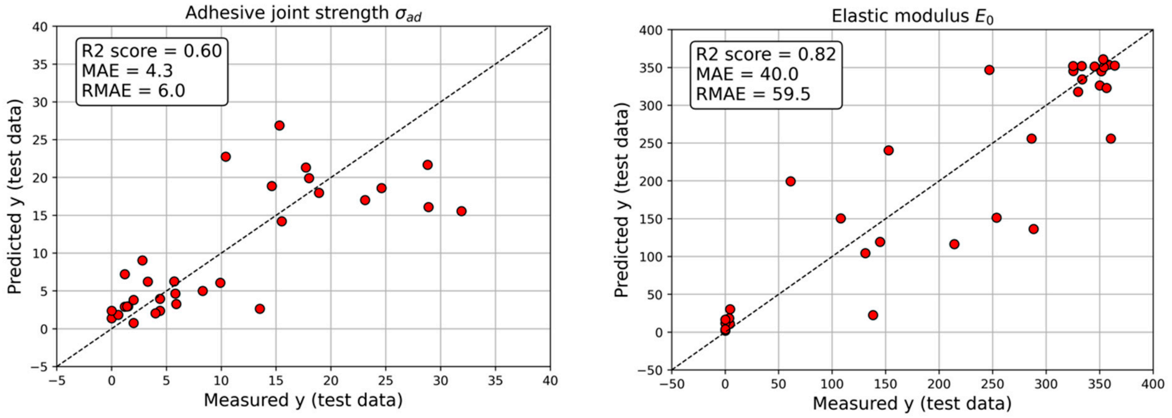

(b) 2nd cycle of active learning loop

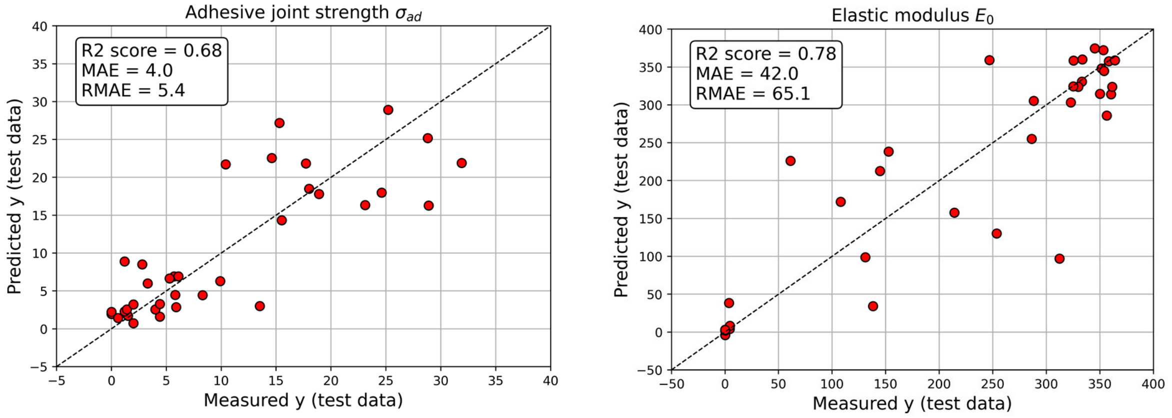

(c) 3rd cycle of active learning loop

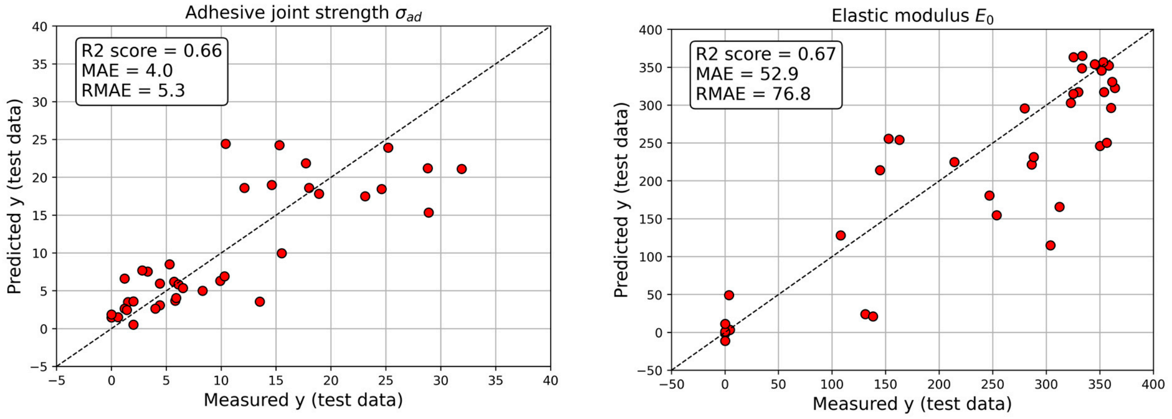

Figure S3. The averaged prediction of adhesive joint strength and elastic modulus from 32-folds cross-validation compared to its measured results from experiment as well as the accuracies of each active learning cycle; (a) 1st cycle, (b) 2nd cycle and (c) 3rd cycle.

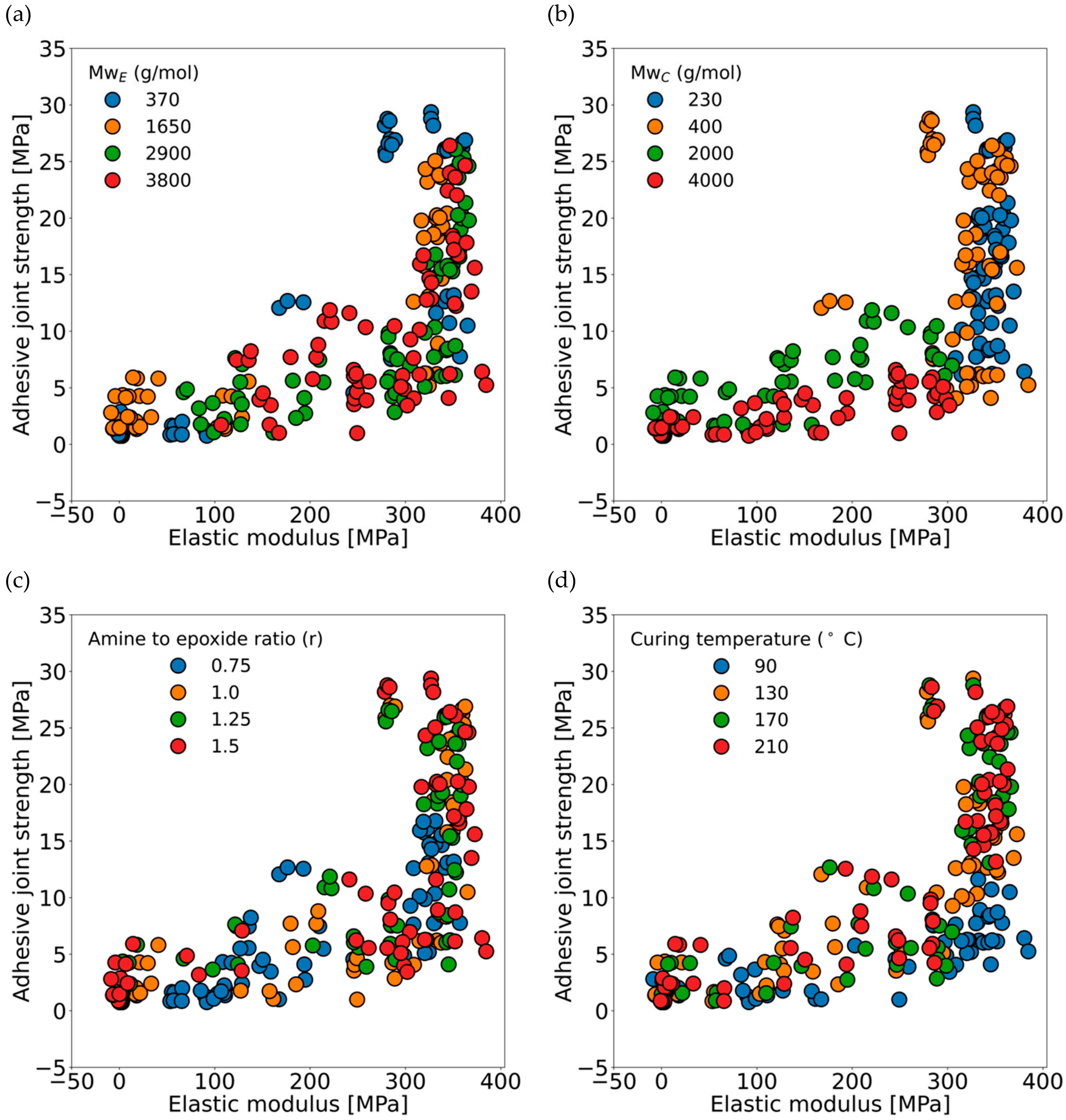

Figure S4. The influence of each variable parameter on the prediction of 256 conditions from the 3rd active learning cycle (a) molecular weight of epoxy resin, (b) molecular weight of curing agent, (c) amine to epoxide ratio and (d) curing temperature.

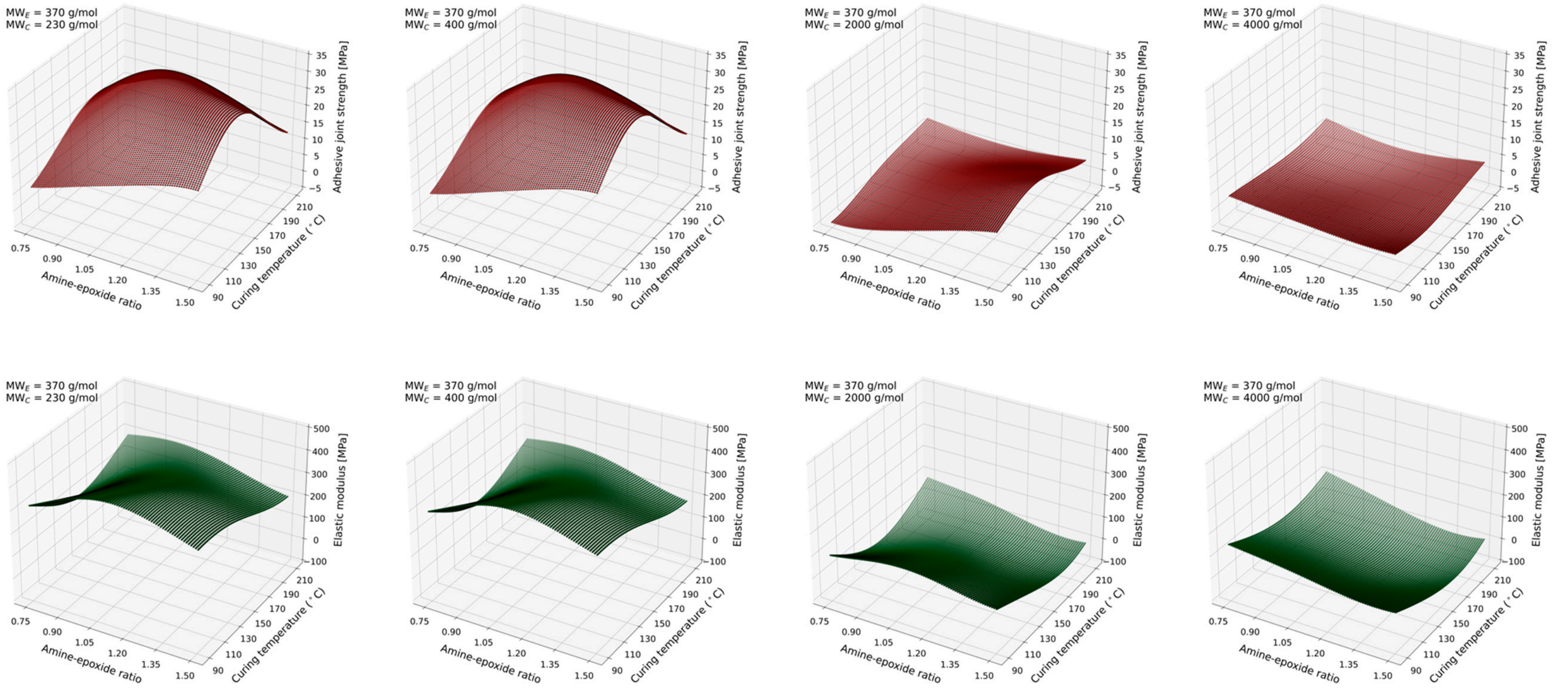

Figure S5. The proposed conditions from Bayesian optimization for adhesive joint strength (MPa) and elastic modulus (MPa) by varying three variable parameters; molecular weight of Jeffamine™ (g/mol), amine-to-epoxide ratio and curing temperature (°C), with the molecular weight of DGEBA at 370 g/mol.

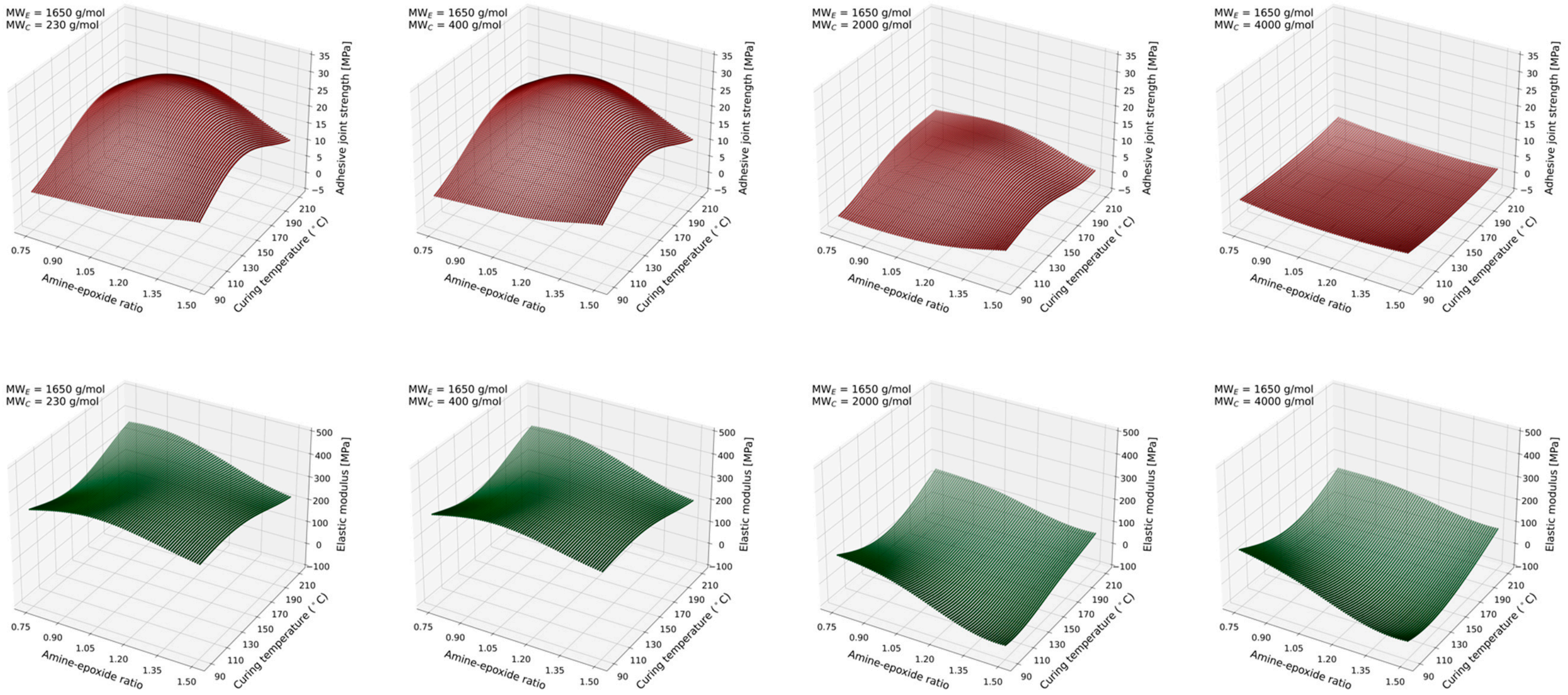

Figure S6. The proposed conditions from Bayesian optimization for adhesive joint strength (MPa) and elastic modulus (MPa) by varying three variable parameters; molecular weight of Jeffamine™ (g/mol), amine-to-epoxide ratio and curing temperature (°C), with the molecular weight of DGEBA at 1650 g/mol.

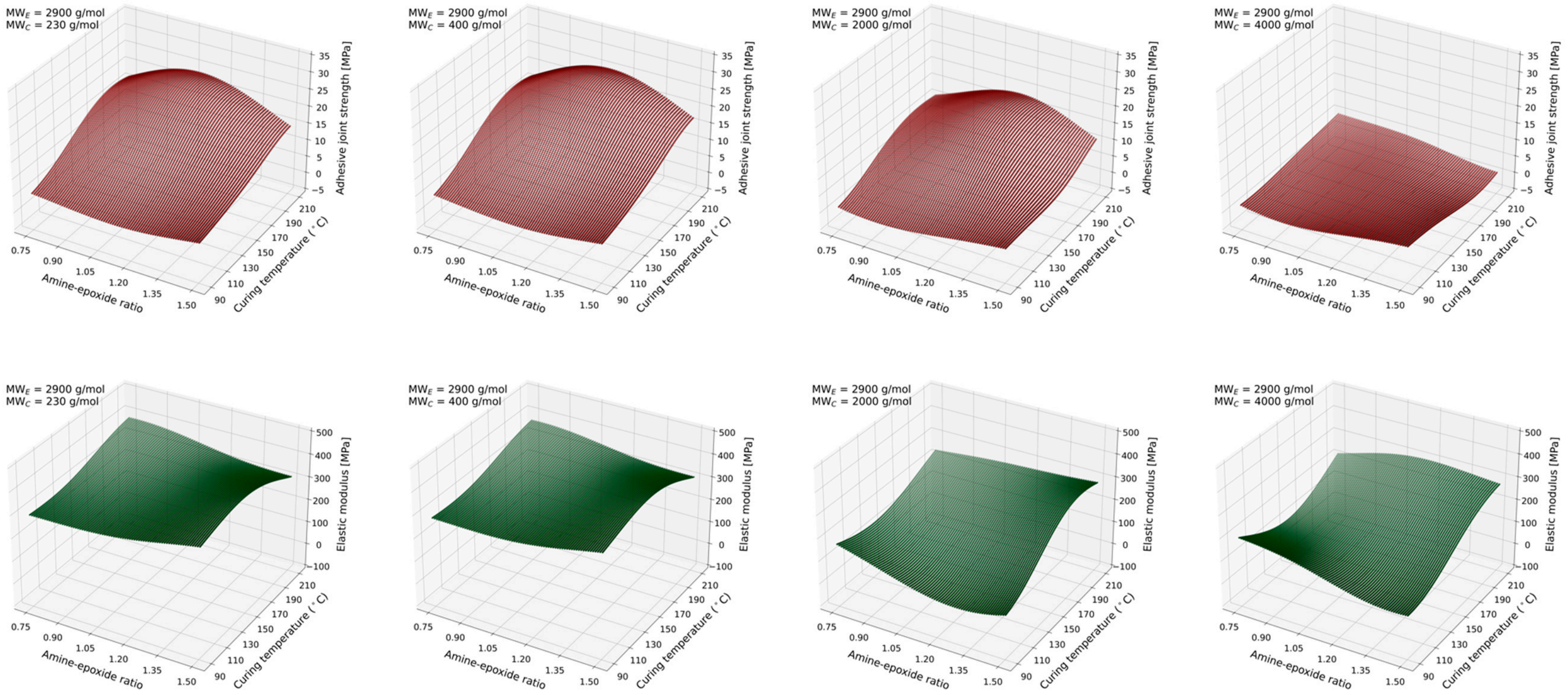

Figure S7. The proposed conditions from Bayesian optimization for adhesive joint strength (MPa) and elastic modulus (MPa) by varying three variable parameters; molecular weight of Jeffamine™ (g/mol), amine-to-epoxide ratio and curing temperature (°C), with the molecular weight of DGEBA at 2900 g/mol.

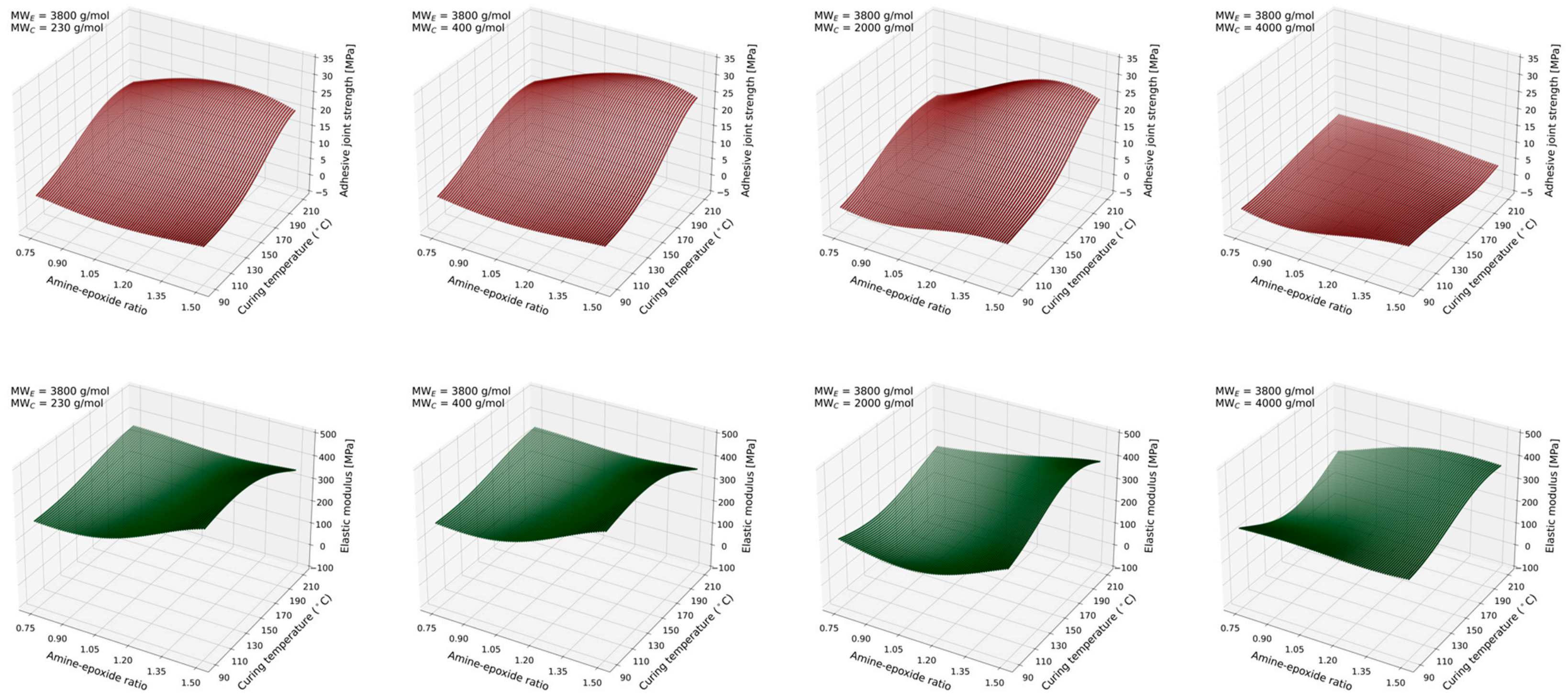

Figure S8. The proposed conditions from Bayesian optimization for adhesive joint strength (MPa) and elastic modulus (MPa) by varying three variable parameters; molecular weight of Jeffamine™ (g/mol), amine-to-epoxide ratio and curing temperature (°C), with the molecular weight of DGEBA at 3800 g/mol.

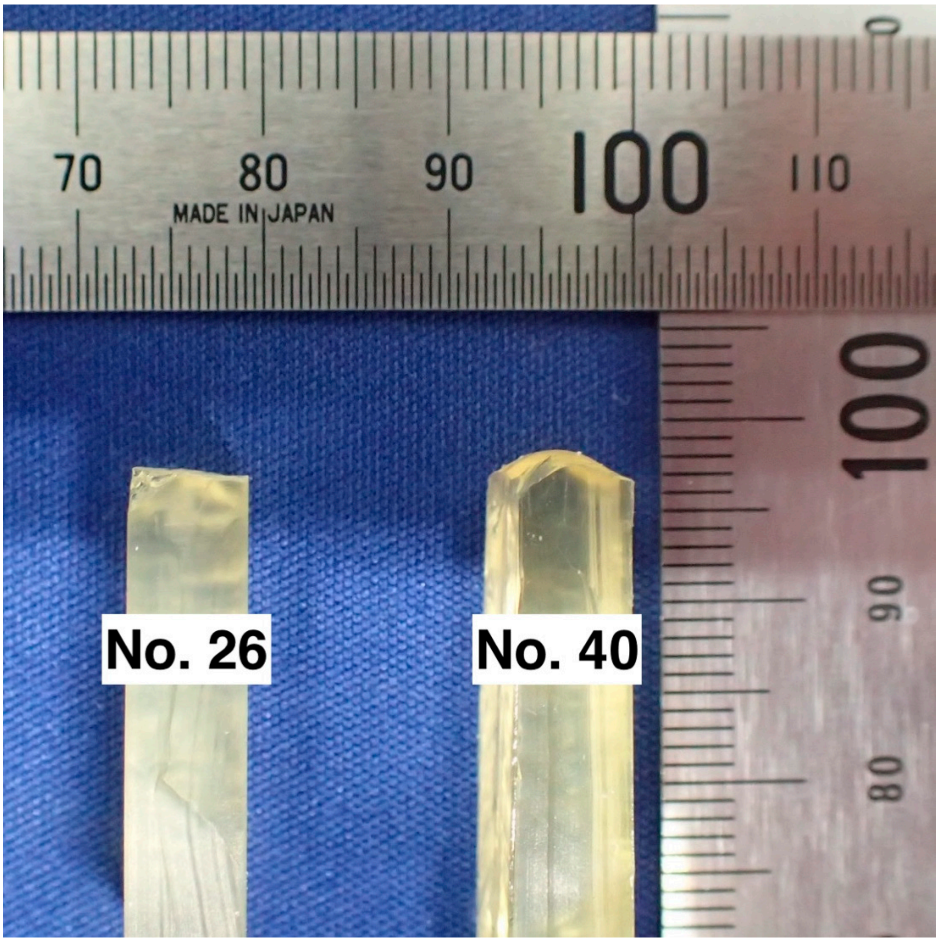

Figure S9. The appearance of specimens after tearing by tensile force of the specimen with condition number 26 (high elastic modulus) shows less ductile behavior compared to the specimen with condition number 40 (less elastic modulus).

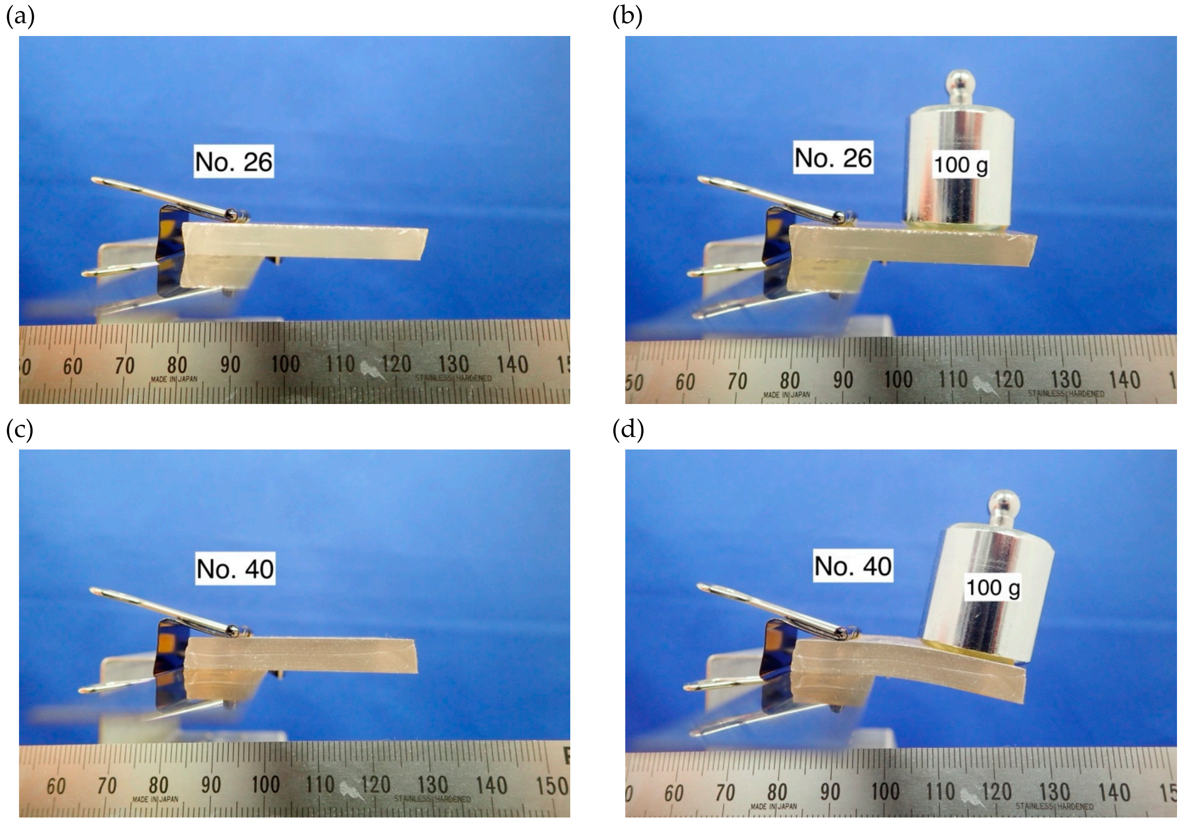

Figure S10. Demonstration of the elasticity behavior of the specimens (a) high elastic modulus specimen no. 26 before adding load, (b) after adding 100 g load, (c) low elastic modulus specimen no. 40 before adding load and (d) after adding 100 g load.

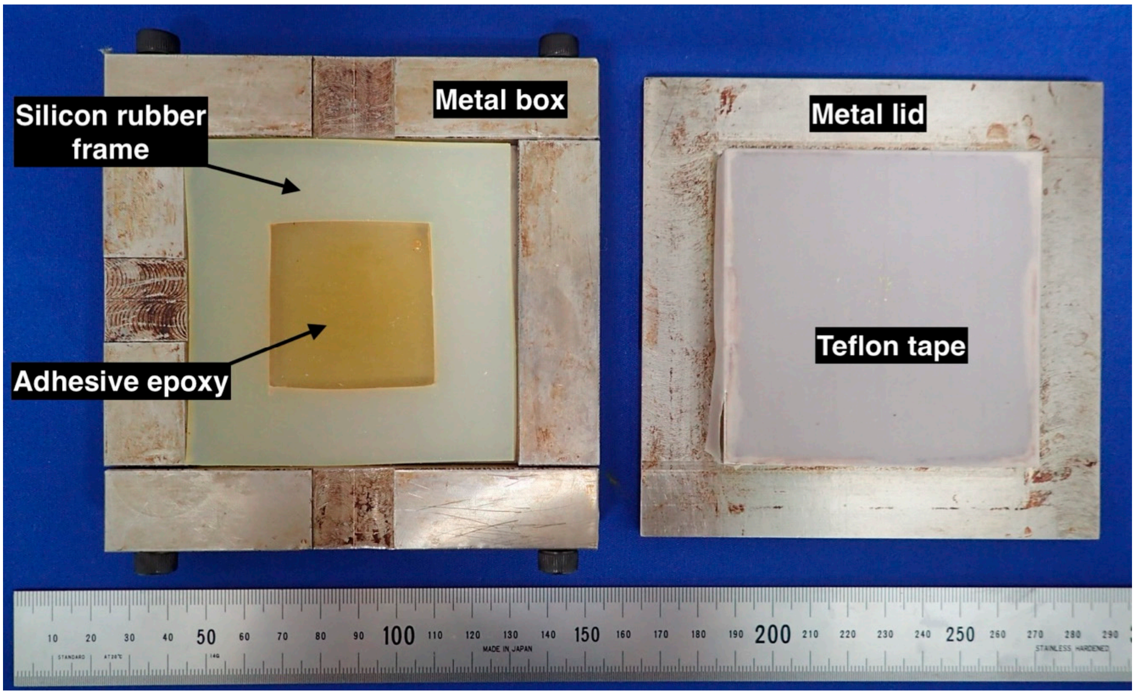

Figure S11. A picture of actual metal mold consists of metal lid, Teflon tape, metal box, silicon rubber frame and adhesive epoxy specimen.

References

1. Standard Test Method for Rubber Property-Durometer Hardness. *American Society for Testing and Materials (ASTM)* 2021.
2. Pruksawan, S.; Lambard, G.; Samitsu, S.; Sodeyama, K.; Naito, M. Prediction and Optimization of Epoxy Adhesive Strength from a Small Dataset through Active Learning. *Sci Technol Adv Mater* **2019**, *20*, 1010–1021, doi:10.1080/14686996.2019.1673670.
